# The Impact of COVID-19 on Amputation and Mortality Rates in Patients with Acute Limb Ischemia: A Systematic Review and Meta-Analysis

**DOI:** 10.3390/diseases12040074

**Published:** 2024-04-07

**Authors:** Lelio Crupi, Alessio Ardizzone, Fabrizio Calapai, Sarah Adriana Scuderi, Filippo Benedetto, Emanuela Esposito, Anna Paola Capra

**Affiliations:** 1 Department of Chemical, Biological, Pharmaceutical and Environmental Sciences, University of Messina, Viale Ferdinando Stagno D’Alcontres, 31, 98166 Messina, Italy; lelio.crupi@unime.it (L.C.); aleardizzone@unime.it (A.A.); fabrizio.calapai@unime.it (F.C.); sarahadriana.scuderi@unime.it (S.A.S.); annapaola.capra@unime.it (A.P.C.); 2 Department of Clinical and Experimental Medicine, University of Messina, 98125 Messina, Italy; 3 Unit of Vascular Surgery, Department of Biomedical and Dental Sciences and Morphofunctional Imaging, Policlinico G. Martino, University of Messina, 98125 Messina, Italy; filippo.benedetto@unime.it

**Keywords:** acute limb ischemia (ALI), amputation, mortality, COVID-19, SARS-CoV-2, meta analysis

## Abstract

Since the inception of the SARS-CoV-2 pandemic, healthcare systems around the world observed an increased rate of Acute Limb Ischemia (ALI) in patients with a COVID-19 infection. Despite several pieces of evidence suggesting that COVID-19 infection may also worsen the prognosis associated with ALI, only a small number of published studies include a direct comparison regarding the outcomes of both COVID-19 and non-COVID-19 ALI patients. Based on the above, a systematic review and a meta-analysis of the literature were conducted, evaluating differences in the incidence of two major outcomes (amputation and mortality rate) between patients concurrently affected by COVID-19 and negative ALI subjects. PubMed (MEDLINE), Web of Science, and Embase (OVID) databases were scrutinized from January 2020 up to 31 December 2023, and 7906 total articles were recovered. In total, 11 studies (*n*: 15,803 subjects) were included in the systematic review, and 10 of them (15,305 patients) were also included in the meta-analysis. Across all the studies, COVID-19-positive ALI patients experienced worse outcomes (mortality rates ranging from 6.7% to 47.2%; amputation rates ranging from 7.0% to 39.1%) compared to non-infected ALI patients (mortality rates ranging from 3.1% to 16.7%; amputation rates ranging from 2.7% to 18%). Similarly, our meta-analysis shows that both the amputation rate (OR: 2.31; 95% CI: 1.68–3.17; *p* < 0.00001) and mortality (OR: 3.64; 95% CI: 3.02–4.39; *p* < 0.00001) is significantly higher in COVID-19 ALI patients compared to ALI patients.

## 1. Introduction

Since its original outbreak in Wuhan at the end of 2019, the Coronavirus Disease 19 (COVID-19) has affected around 770 million people worldwide, claiming, as of October 2023, more than 6.9 million lives [1]. 

The etiological agent responsible for COVID-19 has been identified in the Severe Acute Respiratory Syndrome Coronavirus 2 (SARS-CoV-2) [2].

This pathogen is an enveloped positive-sense single-stranded RNA Betacoronavirus (family Coronaviridae; subfamily Orthocoronavirinae) [3,4].

SARS-CoV-2 is highly pathogenic in nature, and it is transmitted from human to human mainly through respiratory droplets [5]. The virus enters host cells by binding to the human angiotensin-converting enzyme 2 (ACE2) receptor, which is highly expressed in the respiratory tract, especially in the airway epithelial cells and in type 2 pneumocytes [6]. The organism responds to the viral infection with a quick proinflammatory innate response, followed by the activation of adaptive immunity [7]. Subjects affected by COVID-19 may show clinical manifestations of variable severity, from a completely asymptomatic or mildly symptomatic disease (usually characterized by bland respiratory symptoms, fever, and myalgia) to more severe infections requiring hospitalization, and often leading to further complication and death [8]. In this regard, it has been observed that the majority of younger and/or healthy subjects tend to be affected by milder forms of infection, whereas both elderly and patients with comorbidities are disproportionally affected by more severe and lethal forms [9]. A large body of evidence suggests an excessive innate immune response to be responsible for the pathogenesis of several severe symptoms from COVID-19 [10].

Critical COVID-19 infection may primarily affect the lungs (i.e., acute respiratory distress syndrome, pneumonia) [11], or have an extrapulmonary origin (often thromboembolic) [12]. For several of these very dire clinical pictures, it was assumed an interplay between aberrant secretion of cytokines and proinflammatory factors [13] and the onset of sepsis, widespread organ damage, including endothelial damage, and the promotion of a state of coagulopathy [12]. The latter is, in turn, a major driver for pulmonary and systemic thromboembolic events, such as pulmonary embolism, ischemic stroke, and acute limb ischemia (ALI) [14].

ALI is a medical emergency characterized by a sudden significant decrease in blood flow to an extremity [15], mostly as a result of thrombotic or embolic events or due to traumatic causes [16]. Its incidence has been estimated to be around 10–15 cases per 100,000 individuals per year in the general population [16], this figure includes non-traumatic forms, both of thrombotic or embolic origin. ALI can occur in both the upper and lower limbs, the latter being by far the more prevalent accounting for more than 80% of total cases of limb ischemia [17,18] and clinically relevant form [19]. The prognosis of this condition is poor both for the affected limb and for the patient, with an estimated one-year rate of unfavorable outcomes (death/major amputation) as high as 46.6% for lower ALI [20].

Rutherford’s ALI classification divides affected limbs into (i) viable (Grade I); (ii) threatened (Grade IIa and IIb); or (iii) irreversibly damaged (Grade III), according to physical examination (coldness and/or cyanosis of the involved limb), clinical manifestations (i.e., sensory loss, pain, myalgia), and objective findings, such as Doppler flow signals [21].

It has been suggested that patients with COVID-19-induced ALI may suffer an increased rate of negative outcomes, (i.e., higher amputation rates and mortality) [22], and that ALI may significantly affect even younger patients with a lower burden of comorbidities [23].

Although several published articles focused on the association between ALI and COVID-19, many of them, both observational studies and case series, lack a comparison with patients not affected by the viral infection. Moreover, the absence of relevant sample sizes in studies including a control group often hinders the possibility of performing robust comparative analysis. On these bases, we evaluated clinical differences in outcomes between ALI patients with or without COVID-19 infection. For this purpose, we conducted a systematic review and meta-analysis of the literature, dissecting PubMed (MEDLINE), Web of Science, and Embase (OVID) scientific databases to quantitively assess existing differences in amputation and mortality rates between COVID-19 positive and negative ALI patients.

## 2. Methods

### 2.1. Search Strategy

We used the bibliographic databases Web of Science, Embase (OVID), and PubMed (MEDLINE) to conduct the literature search. To describe a thorough search strategy of the papers, we followed the Preferred Reporting Items for Systematic Review and Meta-Analysis Protocols (PRISMA-P) criteria. AA and LC carried out the bibliographic search using only English-language literature and the qualifying criteria indicated in Table 1.

Two content specialists (APC and EE) supervised both the creation of the search strategy and the research. As COVID-19 was declared a public health emergency by the WHO on 30 January 2020 [24], articles published from 30 January 2020 up to 31 December 2023 were included. No specific geographical restriction criteria were imposed. All articles dealing with ALI patients who tested positive for COVID-19 at admission or during their hospitalization, as well as patients treated for COVID-19 who developed ALI during or shortly after the infection (<15 days from the last COVID-19 positive tests), were included.

In either case, we only selected articles that both (a) reported ALI-related amputation and mortality rates and (b) included a comparison group of patients not affected by COVID-19 and who developed ALI. The specific keywords used for the search are listed in Table 2, and include a combination of terms linked to amputation and ALI in the context of COVID-19.

### 2.2. Study Selection

Using the PubMed (MEDLINE), Embase (OVID), and Web of Science databases, we conducted our research to detect records. Then, we eliminated duplicate entries. Subsequently, the two review authors (AA and LC) looked over the titles and abstracts of every finding to eliminate any inappropriate articles. After this step, we scrutinized articles entirely to identify those that fulfilled our eligibility criteria. Including a third review author (APC) helped to reconcile conflicting viewpoints.

Two authors retrieved the data from the included studies (AA and LC), in particular, we gathered the following data: study participants, study catchment area (i.e., geographic zone), publication year, title, author(s), and related clinical outcomes.

### 2.3. Assessment of Risk of Bias

Using the Newcastle Ottawa scale (NOS; see Supplementary Table S1), two reviewers AA and LC independently evaluated the quality of the records included in this systematic review.

Based on the NOS score, the studies were judged as low, medium, or high risk of bias, as previously done in [25,26]. A third review author’s intervention (APC) settled disagreements about score assignments. The following ranges were used as standard: score < 4 “high risk of bias”, a score between 4–6 “intermediate risk of bias”, and a score between 6–9 “low risk of bias”. Following the authors’ assessments, only 1 of the papers were found to be at an “intermediate risk of bias”; the others were assessed as having “low risk of bias”. Judgments of each study are reported in Supplementary Table S1.

### 2.4. Data Synthesis Methods for Meta-Analysis

We used the random-effects model using the Mantel–Haenszel technique and an odds ratio (OR) measure for statistical analysis in the meta-analysis. Estimates of the variant effect (OR) and the associated 95% confidence interval (CI) were effectively merged. The heterogeneity index was assessed using the I^2^ statistic. Review Manager (RevMan Version 5.4., The Nordic Cochrane Centre, The Cochrane Collaboration: Copenhagen, Denmark, 2014) was used to conduct the meta-analysis of the pooled data.

## 3. Results

### 3.1. Findings from Systematic Search

Using the previously mentioned search strategy, we were able to get 7906 publications from the PubMed (MEDLINE), Web of Science, and Embase (OVID) databases; 6576 articles remained after duplicates were removed. By looking at the abstract and title of each article, we were able to check its eligibility; thereafter, 6538 articles were deemed irrelevant, while 38 articles were evaluated in full-text form.

The reasons for the exclusion of the articles during this phase were that they did not match the inclusion criteria, as they did not include a control group consisting of COVID-19 negative ALI patients, did not fit into the required research typology, or did not report clear values regarding amputation and mortality correlated to ALI in COVID-19 positive patients.

**Figure 1:**
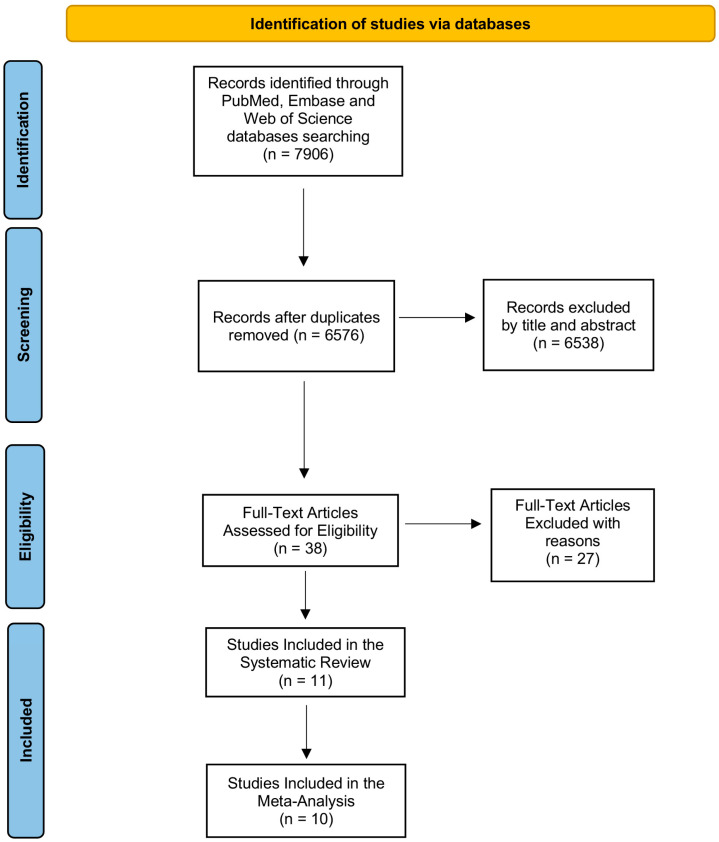
PRISMA flow diagram. Beginning with the identification stage and ending with the final stage that reports the included articles for both systematic review and meta-analysis, the figure depicts the articles that were found in each step of the search strategy.

Ultimately, as shown in Figure 1, 11 publications that met the inclusion criteria were added to this systematic review; from these, 10 records were also employed for the meta-analysis, since they reported data of COVID-19 ALI patients in comparison with a COVID-19 negative ALI group.

### 3.2. Description of Included Studies in the Systematic Review

The systematic review of the literature collected 11 different studies, for a total of 15,803 patients. Seven of these were retrospective studies [22,27,28,29,30,31,32], three were prospective [33,34,35], while one [36] was a prospective study that included a comparison group formed by a retrospectively collected cohort.

Across all the studies, patients affected simultaneously by COVID-19 and ALI were hospitalized between January 2020 and December 2021.

Five studies [27,29,31,32,36] included as a comparison group a cohort of pre-COVID patients, who had been treated for ALI in the period between 2017 and 2019. Four further studies, [22,30,33,34] considered for comparisons negative patients treated concurrently as COVID-19-positive patients. The study by Goldman et al. [27] included both a pre-COVID-19 group and patients who tested SARS-CoV-2 negative during the period from January 2020 to April 2020. Lastly, the study by Indes et al. [28] included in the COVID-19 negative group both patients with a negative Polymerase Chain Reaction (PCR) test and patients without clinical suspicion of infection (not serologically tested).

A high range of geographical heterogeneity was found throughout the studies, as they were conducted across five different continents (five in North America [22,27,28,29,32], three in Europe [34,35,36], one in South America [33], one in Africa [30], and one in Asia [31]).

More than one half of the studies (7 out of 11) included patients admitted to single specialized/tertiary vascular or trauma medical centers [27,28,29,30,33,35,36], whereas in one study, data were collected from a network of four regional vascular surgery hubs [34]. In three further studies, data were extracted from electronic medical records from networks of healthcare organizations [22,32] or obtained from medical records of two tertiary vascular centers [31].

COVID-19 positivity was diagnosed or confirmed in most of the studies [27,28,29,30,31,33,35] with PCR upon admission for ALI to the vascular unit. In two studies, the infection was determined both with Nasopharyngeal swab and Chest X-rays [34,36]. It is worth noting that one of these studies also [36] included 3 patients (out of 15) becoming infected only after hospitalization. In the two retrospective studies conducted on electronic medical records, inclusion criteria were COVID-19 diagnosis was performed <7 days before and up to a day after ALI occurrence [22] and positivity < 14 days before the day of hospitalization for ALI [32], respectively.

In five of the included articles, [30,31,33,34,35] ALI severity was graded according to Rutherford’s ALI classification. This diagnostic classification divides affected limbs into (i) viable (Grade I); (ii) threatened (Grade IIa and Iib); or (iii) irreversibly damaged (Grade III), according to physical examination (coldness and/or cyanosis of the involved limb), clinical manifestations (i.e., sensory loss, pain, myalgia), and objective findings, such as Doppler flow signals [37,38,39]. A single study [31] specifically focused on patients with Grade II ALI.

One of the studies, by Malkoc et al. [29], significantly differed from the others from a methodological standpoint, as it specifically focused on differences in the incidence of thrombotic and ischemic events and amputation rates in patients admitted to an intensive care unit (ICU), both from 2019 (pre-COVID-19 patients, *n*: 249) and from 2020 (patients with a diagnosis of COVID-19, *n*: 249). In this study, out of the 249 COVID-19 patients, 12 of them (4.81%) developed gangrene during hospitalization, compared to a single pre-COVID patient (0.40%) who developed gangrene (resolved without any major negative outcome occurring). Among the 12 COVID-19 patients, the amputation rate was as high as 33.4%, while the mortality rate was equal to 42%. Based on the methodological differences of this article compared to the remaining ones, it will not be included in the subsequent presentation of the results.

Criteria used to define the outcomes (mortality and amputation rates) were different across the other 10 articles. In particular, in four studies, outcomes were expressed as 30-day rates [28,32,33,35], in a single study [22] they were measured as 180-day rates, and in the remaining articles [27,29,30,31,34,36] the occurrence of amputation and mortality were considered present if they occurred during the entire length of the hospitalization.

Mortality rates were reported in all of the 10 studies considered. Compared to patients without the viral infection, a higher death percentage in ALI patients admitted with a COVID-19 infection or developing the disease during hospitalization was observed across all the studies. The above difference was estimated to be statistically significant (*p* < 0.05) in 6/11 studies [22,28,32,33,34,35]. The mortality rate varied from 6.7% [29] up to 47.2% [32] in COVID-19 positive ALI patients (range in COVID-19 negative ALI patients: 3.1–16.7% [27,32,33].

Likewise, amputation rates were reported in 8/10 studies (Kahlberg et al. [34] and Mascia et al. [36] did not provide this information). All eight articles provided data in terms of affected patients, except for the study by Xie et al. [32], which only reported the “number of affected limbs”.

Across all studies, the percentage of patients/limbs subjected to amputation was higher in COVID-19-positive ALI patients than in negative ALI patients. Amputation rates were reported to be significantly higher (*p* < 0.05) in COVID-19 patients compared to non-infected subjects in 4/8 articles [22,32,33,35]. The amputation rate ranged from 7.0% [22] up to 39.1% [33] in COVID-19 positive ALI patients (range in COVID-19 negative ALI patients: 2.7–18% [22,30].

In the remaining articles, both for death and amputation rates, no statistical significance was explicitly reported by the authors, or it was provided only after adjusting for covariates.

Among these last, Goldman et al. [27] evaluated the differences in prognosis and clot burden between positive and negative COVID-19 patients who underwent lower extremity CT angiogram for ischemia at a tertiary care medical center. In this study, the authors specified that when adjusting for the history of peripheral vascular disease (PVD), both the combined outcome death/amputation and the amputation rates were significantly higher in COVID-19-positive patients (*p* < 0.001 and *p* = 0.02, respectively)

The study by Mascia et al. [36] included 116 consecutive patients, 31 of them specifically affected by ALI. Fifteen ALI patients were affected by COVID-19 at admission or contracted the disease during hospitalization. The authors observed a total limb salvage rate across all the patients equal to 93.5%. Furthermore, two patients (one COVID-19 infected) perished during the hospitalization.

In the study conducted by Naouli et al. [30], COVID-19 patients showed a mortality rate and amputation rate of 27.3% and 22.3%, respectively. By comparison, patients without COVID-19 had a mortality rate of 11.5%, and their amputation rate was 18.0%.

Lastly, in the study by Nwilati et al. [31], patients affected by COVID-19 had a higher amputation rate compared with patients without COVID-19 (36.4% vs. 9.4%). Similarly, an increased mortality rate was found in COVID-19 patients when compared to the negative group (36.4% vs. 9.4%).

Collected data from the articles included in this systematic review are summarized in Table 3.

### 3.3. Meta-Analysis

#### 3.3.1. Evaluation of ALI-Related Amputation in COVID-19 Patients

First, we evaluated the recurrence of amputation following ALI in COVID-19 patients. Studies of Mascia [36], Kahlberg [34], and Xie and colleagues [32] were excluded from this analysis, since they did not clearly express this outcome.

The seven articles included showed an ORs range between 1.34–10.33, and there was not an estimated degree of heterogeneity between the studies (I^2^ = 0%).

Additionally, the pooled data analysis showed a statistically significant (*p* < 0.00001) increase in the incidence of amputations among COVID-19 subjects with ALI (OR: 2.31; 95% CI: 1.68–3.17).

**Figure 2:**
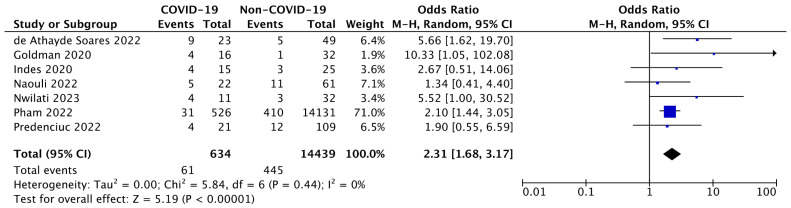
Forest plot of estimating the amputation risk in COVID-19-ALI patients [22,27,28,30,31,33,35]. The blue squares display the effect estimate (ORs), with the size of each blue square corresponding to the weight given to each study in the meta-analysis. Horizontal lines or black arrow represent the 95% CIs corresponding to each effect estimate. The black diamond represents the overall effect of intervention, with its width representing the overall 95% CI. The I^2^ statistic represents a measure of heterogeneity. Overall effect OR: 2.31 [1.68, 3.17]; *p* < 0.00001.

Hence, compared to those without COVID-19, the probability of amputation rose by 131% when ALI developed in the context of SARS-CoV-2 infection as shown in Figure 2.

#### 3.3.2. Evaluation of the Mortality Rate Following ALI in COVID-19 Patients

With a reported death rate of 15–20%, ALI typically develops into advanced limb ischemia within two weeks of its acute onset and presents a significant clinical challenge for vascular surgeons [16].

Considering the scenario of SARS-CoV-2 infection concerning all of these presumptions, in this quantitative estimation we found a considerable mortality rate of COVID-19-ALI subjects with a total OR of 3.64 (95% CI: 3.02–4.39; *p* < 0.00001). Also, in this assessment, the degree of heterogeneity was negligible (I^2^ = 0%).

**Figure 3:**
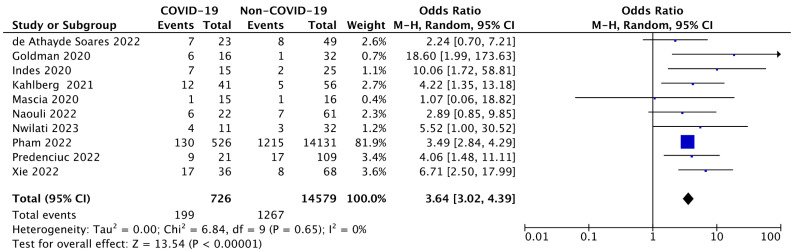
Forest plot of estimating the mortality rate in COVID-19-ALI patients. The blue squares display the effect estimate (ORs), with the size of each blue square corresponding to the weight given to each study in the meta-analysis [22,27,28,30,31,32,33,34,35,36]. Horizontal lines or black arrow represent the 95% CIs corresponding to each effect estimate. The black diamond represents the overall effect of intervention, with its width representing the overall 95% CI. The I^2^ statistic represents a measure of heterogeneity. Overall effect OR: 3.64 [3.02, 4.39]; *p* <0.00001.

This result revealed a powerful statistical significance, indicating the markedly increased mortality rate of ALI patients when presenting COVID-19 compared with non-infected ones. Overall, it can be assumed that the viral infection in addition to aggravating the risk of amputation can also impact patients’ death. Forest plot is shown in Figure 3.

## 4. Discussion

SARS-CoV-2 is a positive-sense single-stranded RNA virus belonging to the Coronaviridae family [40], identified in late 2019 in Wuhan and later spread wildly infecting millions of people worldwide [41], forcing governments to implement lockdown restrictions to control and limit ongoing disease transmission [42].

The rapid viral replication causes an aberrant inflammatory response that can be associated with respiratory and systemic consequences [43]. In addition, it has been assumed that the immune response, triggered by original infection or hidden viral persistence, may result in harmful conditions such as autoimmune symptoms, activation of the coagulation and fibrosis pathways, or metabolic issues [44]. SARS-CoV-2 infection is associated with a high incidence of thrombosis, cerebrovascular complications, and ALI [45].

The pathogenesis of this prothrombotic state is mediated by several mechanisms, including direct viral invasion of endothelial cells, immune-mediated thrombosis and hypercoagulopathy, and activation of the alternative renin-angiotensin system pathway. These alterations have mainly been observed in symptomatic patients with moderate or severe disease, but they have also been described in asymptomatic infections [45].

Therapeutic strategies for ALI may include different approaches on the basis of the severity of the ischemia, the patient’s frailty, the presence of comorbidities, and the degree of vascular involvement. These include pharmacological treatments (i.e., IV administration of unfractionated heparins, fibrinolytic therapy), surgical procedures, and endovascular interventions [16,46]. Prompt intervention is essential to maintain limb function and prevent amputation, particularly in cases of severe reversible ALI (Grade IIa/IIb) [47].

In patients with severe ALI (Grade III), the adverse systemic sequelae associated with ischemia-reperfusion injury, combined with the reduced probability of successfully preserving a functional limb, often make primary amputation the only viable and beneficial strategy [46].

Mild or severe alteration in coagulation occurs in up to 55% of COVID-19 patients [29]; in this regard, several mechanisms have been proposed that may collectively contribute to the pathophysiology of COVID-19-related coagulopathy and explain possible correlations between this condition and ALI. Among the main ones, it has been suggested that a state of hyperinflammation induced by the viral infection may alter coagulation [48] as a result of a complement hyperactivation [49], neutrophil extracellular traps (NETs) formation, or through a substantial reduction in fibrinolytic activity [50]. In addition, SARS-CoV-2 infection may promote a state of coagulative alteration as a result of a direct or cytokine-mediated interference with vascular endothelial homeostasis [49].

In this regard, abnormalities in coagulation parameters, namely fibrinogen, D-dimers, fibrin degradation product (FDP), and prothrombin time, were found in patients with severe forms of COVID-19 and have been significantly associated with increased mortality [51,52].

While COVID-19 is gradually morphing from an unprecedented global pandemic into a persisting endemic disease [53], the urge to understand the prevalence and impact of its most common complications still persists, intending to further improve health systems response and develop new therapeutic strategies [54].

In this regard, a possible association between severe SARS-CoV-2 infection and ALI in hospitalized patients has been observed, since the early stages of the COVID-19 pandemic [55], with an estimated incidence of ALI of up to 0.3% among hospitalized COVID-19 patients [56], with even higher figures for severe patients [57].

Thus, here we aimed to assess the amputation and mortality outcomes related to ALI in COVID-19 and non-COVID-19 patients. Our analysis has several strengths, including the large sample size with a low proportion of variability in the meta-analysis. The included patients came from 30 countries across 5 continents, which enhances the generalizability of our study.

The clinical outcomes object of our study: amputation and mortality related to ALI in SARS-CoV-2 infected patients were compared with a contemporary group of ALI patients with no SARS-CoV-2 infection in the same center. The meta-analysis was conducted to assess the strength of evidence present on the association of ALI with worse outcomes in COVID-19 disease. To our knowledge, this is the largest study to assess the long-term outcome of ALI.

We have defined that these outcomes in patients with COVID-19 are associated with major amputations and higher mortality.

Worse clinical outcomes may be explained by several potential mechanisms, including larger clots with different compositions, increased clot fragmentation leading to distal embolization, and repeated vessel occlusion [45].

This finding is consistent with the notion that biological alterations associated with COVID-19 correlate with disease severity and would only be severe enough in symptomatic patients, as in this case, because only hospitalized patients were included in our cohort. Patients already hospitalized for COVID-19 have likely more severe disease compared with those who are not.

Some study limitations must be addressed. The basal clinical condition of each patient is an evaluation in which different parameters and variables contribute to a complex clinical picture. The time and management of different treatments and procedures in different centers also represent a source of variability within the study.

In this case, the effect of age and gender was not analyzed, because not all the data needed to proceed with a sub-group analysis were reported in the included studies.

The mechanism underlying these systemic coagulation-related disorders remains to be clarified, as well as the variety of highly affected organs during the acute phase and long-term COVID-19 infection.

Considering this, future studies will be able to better validate these preliminary findings, thus providing a more robust characterization regarding the pathophysiological mechanisms of SARS-CoV-2 infection and its driven signaling pathways in different COVID-19 clinical pictures, likely additional factor contributing to the worse disability and higher risk of mortality.

This is to be able to better define patients at greatest risk of worse complications and provide them with effective therapies.

## 5. Conclusions

In conclusion, the results of this systematic review and meta-analysis demonstrate a significant association between COVID-19 infection and worse severe outcomes (specifically death and amputation) in patients affected by ALI. To the best of our knowledge, this is the largest study to analyze differences in ALI outcomes between patients infected by COVID-19 and non-infected individuals. However, further studies are needed to deeper investigate the underlying pathophysiological mechanisms beyond the higher risk of ALI complications in COVID-19 and to identify effective therapeutic interventions for patients at the highest risk of complications. Moving forward, epidemiological studies are needed to investigate any potential increased incidence of ALI or worse outcomes in patients affected by COVID-19, especially long-COVID-19 patients, monitored in follow-up for a longer time.

## Figures and Tables

**Table 1:** Inclusion and exclusion criteria applied for the literature search.

Inclusion Criteria	Exclusion Criteria
Study types: Clinical Trials, Randomized Controlled Trials, Observational studies, Case-control studies, Cross-sectional studies, Cohort studies. Time range: 30 January 2023 up to 31 December 2023. Articles including both COVID-19 positive and negative subjects clearly reporting amputation and mortality rates related to ALI.	Case reports, editorials, letters, reviews, guidelines, abstracts and paper conferences, systematic reviews and meta-analyses, and ongoing studies. Articles not written in English.

**Table 2:** Keywords combined throughout the search plan.

ALI	ALI-Related Outcomes	COVID-19
“Acute limb ischemia”, ALI, ALLI, “Limb Ischemia”, “Limb ischaemia”, Ischemia, Ischemias, Ischaemia, Ischaemias, “limb hypoperfusion”, “extremities ischemia”, “leg ischemia”, “leg ischaemia”	Amputation, amputations, disarticulation, disarticulations, death, deaths, mortality, mortalities	COVID-19, COVID19, 2019-nCoV, “Coronavirus Disease 2019”, “Coronavirus Disease-19”, SARS-CoV-2, “SARS Coronavirus 2”, “Severe Acute Respiratory Syndrome Coronavirus”, Coronavirus, Coronaviruses

**Table 3:** Summary of the included studies.

First Author and Year of Publication	Type of Study	Patients Included	Differences in Amputation Rate *	Differences in Mortality *	Reference
de Athayde Soares et al., 2022	Prospective cohort study	Total (*n*: 72) - ALI and COVID-19 infection (*n*: 23) - ALI without COVID-19 infection (*n*: 49)	ALI and COVID-19: 9/23 (39.1%) vs. ALI without COVID-19: 5/49 (10.2%)	ALI and COVID-19: 7/23 (30.4%) vs. ALI without COVID-19: 8/49 (16.7%)	[33]
Goldman et al., 2020	Retrospective propensity score-matched study	Total (*n*: 48) - ALI and COVID-19 infection (*n*: 16) - ALI without COVID-19 infection (*n*: 32)	ALI and COVID-19: 4/16 (25.0%) vs. ALI without COVID-19: 1/32 (3.1%) Amputations occurring during the index hospital admission	ALI and COVID-19: 6/16 (37.5%) vs. ALI without COVID-19: 1/32 (3.1%) Death occurring during the index hospital admission	[27]
Indes et al., 2020	Retrospective cohort study	Total (*n*: 40) - ALI and COVID-19 infection (*n*: 15) - ALI without COVID-19 infection (*n*: 25)	ALI and COVID-19: 4/15 (26.7%) vs. ALI without COVID-19: 3/25 (12.0%)	ALI and COVID-19: 7/15 (46.7%) vs. ALI without COVID-19: 2/25 (8.0%)	[28]
Kahlberg et al., 2021	Prospective observational study	Total ALI patients (*n*: 97) - ALI and COVID-19 infection (*n*: 41) - ALI without COVID-19 infection (*n*: 241)	-	ALI and COVID-19: 12/41 (29.3%) vs. ALI without COVID-19: 5/56 (8.9%) In hospital deaths	[34]
Malkoc et al., 2022	Retrospective case-control study	Total (*n*: 498) - ICU—COVID-19 positive (*n*: 249) - ICU—COVID-19 negative (*n*: 249)	12/249 ICU COVID-19 positive patients developed a gangrene during the ICU stay (4.8%), 4/12 (33.4%) underwent a major amputation vs. 1/249 (0.4%) ICU COVID-19 negative patients developed a gangrene during the ICU-stay, 0/1 underwent any amputation.	Mortality among the COVID-19 positive patients that developed gangrene: 5/12 (41.7%) vs. Mortality among the ICU COVID-19 positive patients: 0/1	[29]
Mascia et al., 2020	Prospective/retrospective study	Total (*n*: 31) - ALI and COVID-19 infection (*n*: 15) - ALI without COVID-19 infection (*n*: 16)	Total limb salvage rate, considering both COVID-19 positive and COVID-19 negative patients: 29/31 (93.5%)	ALI and COVID-19: 1/15 (6.7%) vs. ALI without COVID-19: 1/16 (6.2%) Death occurring during hospitalization	[36]
Naouli et al., 2022	Retrospective observational study	Total (*n*: 83) - ALI and COVID-19 infection (*n*: 22) - ALI without COVID-19 infection (*n*: 61)	ALI and COVID-19: 5/22 (22.7%) vs. ALI without COVID-19: 11/61 (18.0%) In hospital amputations	ALI and COVID-19: 6/22 (27.3%) vs. ALI without COVID-19: 7/61 (11.5%) In hospital deaths	[30]
Nwilati et al., 2023	Retrospective cross-sectional study	Total (*n*: 43) - ALI and COVID-19 infection (*n*: 11) - ALI without COVID-19 infection (*n*: 32)	ALI and COVID-19: 4/11 (36.4%) vs. ALI without COVID-19: 3/32 (9.4%) Amputations occurring during the entire length of the hospitalization	ALI and COVID-19: 4/11 (36.4%) vs. ALI without COVID-19: 3/32 (9.4%) Death occurring during the entire length of the hospitalization	[31]
Pham et al., 2022	Retrospective propensity score-matched study	Total (*n*: 14,657) - ALI and COVID-19 infection (*n*: 526) - ALI without COVID-19 infection (*n*: 14,131)	ALI and COVID-19: 31/526 (7.0%) vs. ALI without COVID-19: 381/14,131 (2.7%) 180 days amputation rate	ALI and COVID-19: 130/526 (24.7%) vs. ALI without COVID-19: 1215/14,131 (8.6%) 180 days mortality	[22]
Predenciuc et al., 2022	Prospective observational study	Total (*n*: 130) - ALI and COVID-19 infection (*n*: 21) - ALI without COVID-19 infection (*n*: 109)	ALI and COVID-19: 4/21 (19.0%) vs. ALI without COVID-19: 12/109 (11.0%)	ALI and COVID-19: 9/21 (42.9%) vs. ALI without COVID-19: 17/109 (15.6%)	[35]
Xie et al., 2022	Retrospective cohort study	Total (*n*: 104) - ALI and COVID-19 infection (*n*: 36–*n*: 40) - ALI without COVID-19 infection (*n*: 68–*n*: 74 limbs)	ALI and COVID-19: 11/40 limbs (27.5%) vs. ALI without COVID-19: 2/74 limbs (2.7%)	ALI and COVID-19: 17/36 (47.2%) vs. ALI without COVID-19: 8/68 (11.8%)	[32]

* where not otherwise specified, data are expressed as “30-days amputation rate” and as “30-days days mortality rate”.

## Data Availability

Data of this study are available from the corresponding author’s address.

## Supplementary Materials

The following supporting information can be downloaded at: https://www.mdpi.com/article/10.3390/diseases12040074/s1, Table S1: Risk of bias judgements; Table S2: PRISMA checklist [58]; Figure S1: Funnel plots.
